# The Anxiolytic and Antidepressant Effects of Diallyl Disulfide and GYY4137 in Animals with Chronic Neuropathic Pain

**DOI:** 10.3390/antiox10071074

**Published:** 2021-07-03

**Authors:** Xue Bai, Gerard Batallé, Olga Pol

**Affiliations:** 1 Grup de Neurofarmacologia Molecular, Institut d’Investigació Biomèdica Sant Pau, Hospital de la Santa Creu i Sant Pau, 08041 Barcelona, Spain; xue.bai@e-campus.uab.cat (X.B.); gerard.batalle@e-campus.uab.cat (G.B.); 2 Grup de Neurofarmacologia Molecular, Institut de Neurociències, Universitat Autònoma de Barcelona, 08193 Barcelona, Spain

**Keywords:** anxiety, apoptosis, depression, hydrogen sulfide, neuropathic pain, oxidative stress

## Abstract

When neuropathic pain is maintained long term, it can also lead to the development of emotional disorders that are even more intense than pain perception and difficult to treat. Hydrogen sulfide (H_2_S) donors relieve chronic pain, but their effects on the associated mood disorders are not completely elucidated. We evaluated if treatment with DADS (diallyl disulfide) or GYY4137 (morpholin-4-ium 4-methoxyphenyl(morpholino) phosphinodithioate dichloromethane complex), two slow-releasing H_2_S donors, inhibits the anxiety- and depressive-like behaviors that concur with chronic neuropathic pain generated by sciatic nerve injury in mice. The modulatory role of these drugs in the inflammatory, apoptotic, and oxidative processes implicated in the development of the affective disorders was assessed. Our results revealed the anxiolytic, antidepressant, and antinociceptive properties of DADS and GYY4137 during neuropathic pain by inhibiting microglial activation and the up-regulation of phosphoinositide 3-kinase/phosphorylated protein kinase B and BAX in the amygdala (AMG) and/or periaqueductal gray matter (PAG). Both treatments also normalized and/or activated the endogenous antioxidant system, but only DADS blocked ERK 1/2 phosphorylation. Both H_2_S donors decreased allodynia and hyperalgesia in a dose-dependent manner by activating the Kv7 potassium channels and heme oxygenase 1 signaling pathways. This study provides evidence of the anxiolytic and antidepressant properties of DADS and GYY4137 during neuropathic pain and reveals their analgesic actions, suggesting that these therapeutic properties may result from the inhibition of the inflammatory, apoptotic, and oxidative responses in the AMG and/or PAG. These findings support the use of these treatments for the management of affective disorders accompanying chronic neuropathic pain.

## 1. Introduction

Several studies have demonstrated that the prevalence of neuropathic pain in the general population is around 6.9–10% [1]. It is also well known that, when neuropathic pain is maintained long term, in addition to the symptoms of the pain itself, it is very common to develop emotional disorders that are even more intense than pain perception, making their treatment a challenge [2]. Current treatments reduce pain symptoms but have limited efficacy in reducing the mood disorders that co-occur with neuropathic pain; consequently, new therapies are urgently needed.

Hydrogen sulfide (H_2_S) is a gaseous neurotransmitter that regulates numerous physiological and pathophysiological processes [3]. It is highly implicated in modulating the cellular redox state and protects cells from oxidative stress [4,5]. Previous works reported the anxiolytic and/or antidepressant effects of fast exogenous H_2_S donors such as sodium hydrosulfide (NaHS) [6] and sodium sulfide (Na_2_S) [7] and the improvement of the anxiety- and/or depressive-related behaviors accompanying diabetes [8,9,10]. In addition, the antidepressant effects of H_2_S donors that could release H_2_S in a more controlled manner, such as diallyl disulfide (DADS), a garlic component, in animals with depressive-like behaviors induced by chronic mild stress (CMS) have been also demonstrated [11]. GYY4137 (morpholin-4-ium 4-methoxyphenyl(morpholino) phosphinodithioate dichloromethane complex), a synthetic H_2_S donor that also slowly releases H_2_S over a long time period [12], has been also recognized as a promising therapeutic agent in cardiovascular diseases, inflammatory processes, diabetes, and cancer [13]. Nevertheless, the potential effects of DADS and GYY4137 in modulating the emotional disorders associated with chronic pain have not been established.

Microglial activation and the increased synthesis of phosphoinositide 3-kinase (PI3K)/protein kinase B (Akt) induced by nerve injury in the spinal cord and several brain regions provoke central sensitization, thus contributing to the development of anxiety- and/or depressive-like behaviors associated with chronic pain both in animals [14,15] and patients [16]. Microglial activation has been exhibited in the amygdala (AMG) of mice with anxiety-like behaviors, and its blockage inhibited this emotional behavior [17].

Recent studies also reveal the direct involvement of oxidative stress in anxiety- and depressive-like behaviors [18,19]. Oxidative stress, displayed with high levels of reactive oxygen species (ROS) and/or low levels of antioxidant enzymes, such as heme oxygenase 1 (HO-1) and quinone oxidoreductase 1 (NQO1) in the spinal cord, hippocampus, and prefrontal cortex, is also implicated in the maintenance of neuropathic pain triggered by the chronic constriction of the sciatic nerve (CCI) [15,20]. In consequence, the inhibition of the PI3K/p-Akt signaling pathways attenuated neuropathic pain [21,22] and treatment with antioxidants, such as oxindoles and Nrf2 and/or HO-1 inducers, exerted anxiolytic and antidepressant effects by enhancing and/or avoiding the depletion of antioxidant enzymes induced by neve injury or peripheral inflammation [20,23,24]. Earlier studies reveal that NaHS blocked oxidative stress and increased the synthesis of superoxide dismutase (SOD) in the hippocampus of diabetic rats [8]. Similarly, isothiocyanates such as allyl-isothiocyanate (A-ITC) and phenyl-isothiocyanate (P-ITC) also avoided the enhanced expression of p-Akt/AKT or p-ERK ½ and improved the protein levels of HO-1, NQO1, and glutathione S-transferase Mu 1 (GSTM1) in the hippocampus and prefrontal cortex of animals with neuropathic pain [25]. Nevertheless, the effects of DADS and GYY4137 in the nociceptive and oxidative responses provoked by peripheral nerve lesion in the central nervous system have not been assessed.

It is well known that oxidative stress leads to cell apoptosis as manifested with the up-regulation of Bcl2-associated X (BAX), an apoptosis-related protein, in the midbrain of rats with neuropathic pain [26,27]. In this study, the effects of DADS and GYY4137 in the apoptotic reactions generated by nerve injury will be also evaluated.

The AMG is an important brain area involved in the modulation of emotional disorders, such as anxiety and depression, as well as in the development of chronic pain including neuropathic pain [11,28,29,30]. In addition, several direct and indirect pathways between the periaqueductal gray area (PAG) and AMG participating in the modulation of neuropathic pain have also been demonstrated [11,31], thus supporting the participation of these brain areas in the control of neuropathic pain and associated affective disorders.

In this study, we evaluated the anxiolytic and antidepressant effects of DADS and GYY4137 in mice with neuropathic pain induced by CCI. The mechanisms implicated in their analgesic actions and their effects in microglial activation and expression of PI3K/p-Akt, BAX, and several antioxidant enzymes in the AMG and PAG were further investigated.

## 2. Materials and Methods

### 2.1. Animals

Experiments were carried out with male C57BL/6 mice (21–26 g; 5–6 weeks old), acquired from Envigo Laboratories (Barcelona, Spain), which were housed under standard light/dark (12/12-h), temperature (22 °C), and humidity (66%) conditions with free access to food and water. Experiments were performed after 7 days of acclimatization to the environmental conditions, conducted between 9:00 a.m. and 5:00 p.m., and in conformity with the guidelines of the European Commission’s directive (2010/63/EC) and the Spanish Law (RD 53/2013) regulating animal research, and approved by the local Committee of Animal Use and Care of the Autonomous University of Barcelona (ethical code: 9863). Maximal efforts were made to reduce the number and suffering of animals used.

### 2.2. Induction of Neuropathic Pain

Neuropathic pain was provoked by CCI. Sciatic nerve ligation was carried out under isoflurane anesthesia conditions (3% induction, 2.5% maintenance). The biceps femoris and the gluteus superficialis were separated by blunt dissection, and three ligatures right (4/0 silk) around the sciatic nerve were performed taking care to preserve epineural circulation as described by [32]. The same conditions were applied to control animals without nerve ligation (sham).

### 2.3. Mechanical Allodynia

Mechanical allodynia was evaluated by measuring the hind paw withdrawal response after the stimulation with the von Frey filament of different bending forces (0.008–3.5 g). Animals were placed in Plexiglas tubes (20 cm high × 9 cm diameter) with a wire grid bottom through which the filaments (North Coast Medical, Inc., San Jose, CA, USA) were applied by using the up–down paradigm [33]. A filament of 0.4 g was applied first, and the filament of 3.0 g was used as a cut-off. The strength of the next filament was increased or decreased depending on the animal’s response. The threshold of the response was calculated using an Excel program (Microsoft Iberia SRL, Barcelona, Spain) that included curve fitting of the data.

### 2.4. Thermal Hyperalgesia

Thermal hyperalgesia was measured by assessing the paw withdrawal latency in response to radiant heat in the plantar test (Ugo Basile, Varese, Italy) [34]. Mice were placed in Plexiglas tubes (20 cm high × 9 cm diameter) placed on a glass surface. The heat source positioned under the plantar surface of the hind paws was activated with a light beam intensity until the paw withdrawal. We used a cut-off time of 12 s. Mean paw withdrawal latencies were determined from the average of three separate trials.

### 2.5. Cold Allodynia

Cold allodynia was evaluated using the cold plate apparatus (Ugo Basile, Italy). The number of elevations of each hind paw from mice exposed to the cold plate (4 ± 0.5 °C) during 5 min was recorded.

In all tests, animals were habituated to the environment for 1 h before the experiment. Both ipsilateral and contralateral paws were tested.

### 2.6. Anxiety Behavioral Tests

The anxiety-like behavior was assessed by utilizing the elevated plus maze (EPM) [35] and the open file (OF) tests [36]. The EPM has an X-shape with 4 arms each of 5 cm wide and 35 cm long, two open and two closed, with walls 15 cm high. The height from the labyrinth to the ground is 45 cm. The animal was placed in the center of the maze facing the open arms and its behavior was recorded by a digital camera for 5 min. The number of entries into the open and closed arms and the percentage of time stay in the open arms were calculated for each animal.

In the OF test, mice were placed in the center of the arena of a 44 × 44 cm box with a grey non-reflecting base and four walls, and their behavior was recorded by a digital camera for 5 min. Animals were allowed to move freely around the maze and to explore the environment. The number of entries in the central area, the time spent in it and the number of squares crossed was assessed.

### 2.7. Depressive Behavioral Tests

The evaluation of the depressive-like behaviors was performed by using the tail suspension test (TST) and the forced swimming test (FST) in which the duration of immobility of the animals was quantified according to the methods described by [37,38], respectively.

In the TST, mice isolated acoustically and visually were suspended by the tail from a horizontal wooden bar (35 cm above the floor) using adhesive tape (1 cm from the tip of the tail). The immobility time in seconds was recorded for 6 min. In the FST, each mouse was placed in a transparent Plexiglas cylinder (25 cm × 10 cm) containing water to a depth of 10 cm at 24 °C ± 0.1 °C. Each animal was subjected to forced swimming for 6 min, and the total duration of immobility was measured during the last 4 min, when mice show a sufficiently stable level of immobility. In both tests, mice were considered immobile when they remained completely quiet.

All of these experiments were performed by experimenters blinded to the experimental conditions.

### 2.8. Western Blot Analysis

Mice were euthanized by cervical dislocation at 30 days after surgery (CCI or SHAM). The contralateral AMG and the PAG were extracted and preserved at 80 °C until use. We analyzed the expression of PI3K, p-Akt/Akt, p-ERK1/2/ERK1/2, CD11b/c, BAX, HO-1, NQO1, SOD-1, and glutathione S-transferase Mu 1(GSTM1) by Western blot assay. The sonication of tissues was made in cold lysis buffer RIPA Buffer (Sigma-Aldrich). After solubilization for 1 h at 4 °C, crude homogenates were sonicated for 10 s and centrifuged at 700× *g* for 20 min at 4 °C. The supernatant (60 μg of total protein) was mixed with 4X Laemmli loading buffer and loaded onto a 4% stacking/12% separating sodium dodecyl sulfate polyacrylamide gels. Proteins were electrophoretically transferred onto a polyvinylidene fluoride membrane for 120 min and successfully blocked with phosphate-buffered saline (PBS) containing 5% nonfat dry milk, Tris-buffered saline with Tween 20 containing 5% bovine serum albumin (BSA), or 5% nonfat dry milk and PBS with Tween 20 containing 5% BSA for 75 min; they were then incubated with specific rabbit primary antibodies anti PI3K (1:150; Abcam, Cambridge, United Kingdom), phospho-Akt (1:150; Cell Signaling Technology, Danvers, MA, USA), total Akt (1:250; Cell Signaling Technology, Danvers, MA, USA), phospho-ERK 1/2 and total ERK 1/2 (1:250; Cell Signaling Technology, Danvers, MA, USA), BAX (1:250; Cell Signaling Technology, Danvers, MA, USA), HO-1 (1:250; Abcam, Cambridge, UK), NQO1 (1:200; Abcam, Cambridge, UK), SOD-1 (1:150; Novus Biologic, Littleton, CO, USA), and GSTM1 (1:150; Novus Biologic, Littleton, CO, USA) or β-actin (1:5000, Abcam Cambridge, UK) overnight at 4 °C. The blots were then incubated with anti-rabbit secondary polyclonal antibodies conjugated to horseradish peroxidase (GE Healthcare, Little Chalfont, Buckinghamshire, UK) for 1 h at room temperature. Proteins were detected by using chemiluminescence reagents provided in the ECL kit (GE, Healthcare, Little Chalfont, Buckinghamshire, UK). Densitometric analysis was carried out using Image-J program (National Institutes of Health, Bethesda, MD, USA).

### 2.9. Experimental Procedures

In a first set of experiments, we investigated if the repetitive administration of 150 µmols/kg of DADS from day 28 to 30 after surgery or 32 µmols/kg of GYY4137 from day 27 to 30 after surgery (two times per day) inhibited the anxiety- and depressive-like behaviors associated with chronic pain. The effects of these treatments in the mechanical allodynia, thermal hyperalgesia, and cold allodynia provoked by nerve injury were also assessed (*n* = 6–8 animals per group). The dose of DADS and GYY413 was selected in accordance with other studies [11,39].

In other groups of animals, we evaluated the antinociceptive effects produced by the acute administration of different doses of DADS (12.5–200 µmols/kg) and GYY4137 (2–64 µmols/kg) in CCI mice. The reversion of the analgesic effects made by the acute administration of high doses of DADS (200 µmols/kg) or GYY4137 (64 µmols/kg) with 8.0 µmol/kg of the selective Kv7 potassium channel blocker, XE-991, or 14.5 µmol/kg of the HO-1 inhibitor (tin protoporphyrin IX, SnPP) [40] were also evaluated (n = 6 animals per group). In all experiments, sham-operated mice were used as controls.

Sciatic nerve-injured animals treated with DADS, GYY4137, or vehicle (0.9% saline solution, SS) were euthanized by cervical dislocation, and the protein levels of PI3K, p-Akt, p-ERK 1/2, CD11b/c, BAX, HO-1, NQO1, SOD-1, and GSTM1 in the AMG and PAG were evaluated by Western blot. In these experiments, sham-operated mice treated with vehicle were used as controls (*n* = 3–4 samples per group).

### 2.10. Drugs

DADS and GYY4137 acquired from Sigma-Aldrich (St. Louis, MO, USA) were dissolved in SS and intraperitoneally administered in a final volume of 10 ml/kg, 1 h before testing, in accordance with our previous pilot studies and other works [11,41]. XE-911 and SnPP purchased in Tocris Bioscience (Ellisville, MO, USA) and Frontier Scientific (Livchem GmbH & Co., Frankfurt, Germany) were dissolved in dimethyl sulfoxide (1% in SS) and administered via intraperitoneal at 8 µmols/kg and 14.5 µmol/kg in a final volume of 10 mL/kg, 30 min before conducting the behavioral tests in accordance with previous studies [42,43]. All drugs were freshly prepared before use. For each group treated with a drug, the respective control group received the same volume of the corresponding vehicle.

### 2.11. Statistical Analyses

Data are expressed as the mean values ± standard error of the mean (SEM). We used the GraphPad software (version 8.0) for the statistical analysis. A one-way ANOVA followed by the Student–Newman–Keuls test was utilized for evaluating the effects of DADS and GYY4137 administered alone and combined with XE-911 or SnPP. The effects of DADS and GYY4137 in the expression of several proteins were also analyzed by using a one-way ANOVA and the post hoc Student–Newman–Keuls test. The ED_50_ of the drugs was calculated by linear regression analysis. A value of *p* < 0.05 was considered significant.

In the von Frey filaments and plantar tests, antinociception is expressed as the percentage of maximal possible effect, where the test latencies pre-drug (baseline) and post-drug administration are compared and calculated in accordance with the following equation:

Maximal possible effect (%) = [(drug-baseline)/(cut-off-baseline)] × 100(1)

In the cold plate, antinociception is expressed according to the following equation:

Inhibition (%) = [(number of paw elevations at baseline − number of paw elevations after drug)/number of paw elevations at baseline)] × 100.(2)

## 3. Results

### 3.1. Anxiolytic and Antidepressant Effects of DADS and GYY4137 in Animals with Chronic Neuropathic Pain

**Figure 1:**
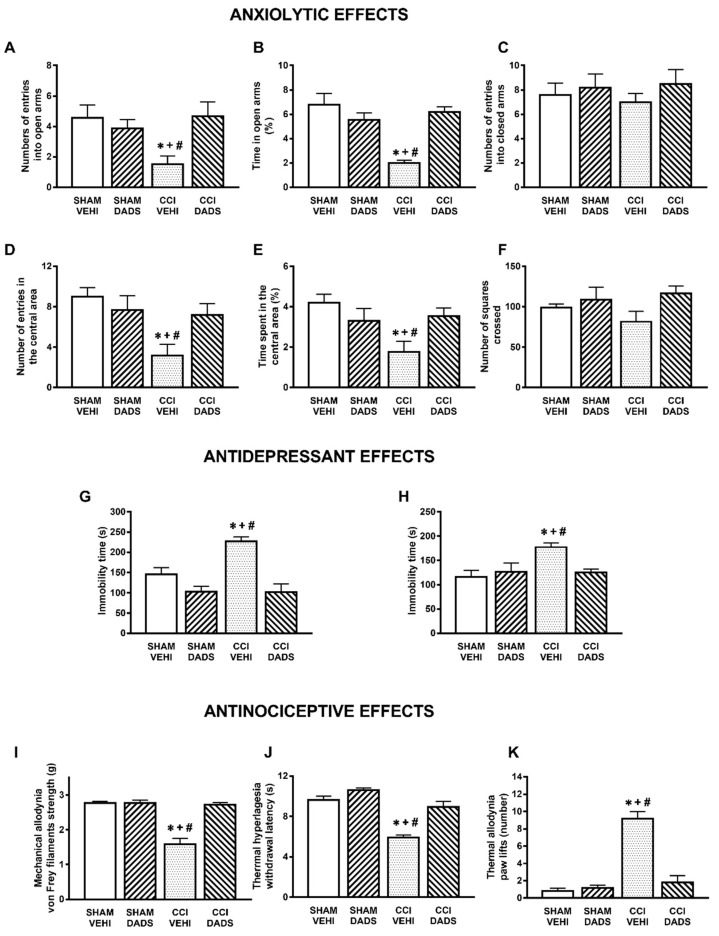
Treatment with DADS inhibited the anxiety- and depressive-like behaviors and the nociceptive responses induced by nerve injury. Effects of the repeated administration with DADS or vehicle at 150 µmol/kg for 3 days, 2 times for each day, on the anxiety-, depressive-, and nociceptive-like behaviors induced by nerve injury at 30 days after surgery. The effects of DADS or vehicle in sham-operated mice are also displayed. In the EPM test, the number of entries to the open arms (**A**), percentage of the time spent in the open arms (**B**), and the number of entries into the closed arms (**C**) are shown. In the OF test, the number of entries in the central area (**D**), the percentage of time spent in the central area (**E**), and the number of squares crossed (**F**) are presented. In the TST (**G**) and FST (**H**), the immobility times (s) are shown. The effects of DADS in the mechanical allodynia (**I**), thermal hyperalgesia (**J**), and thermal allodynia (**K**) in the ipsilateral paw of sham-operated or sciatic nerve-injured mice are also indicated. For each test: * denotes significant differences vs. sham-operated mice treated with vehicle, + vs. sham-operated mice treated with DADS, and # vs. CCI mice treated with DADS (*p* < 0.05; one-way ANOVA followed by the Student–Newman–Keuls test). Mean values ± SEM; *n* = 6–8 animals.

**Figure 2:**
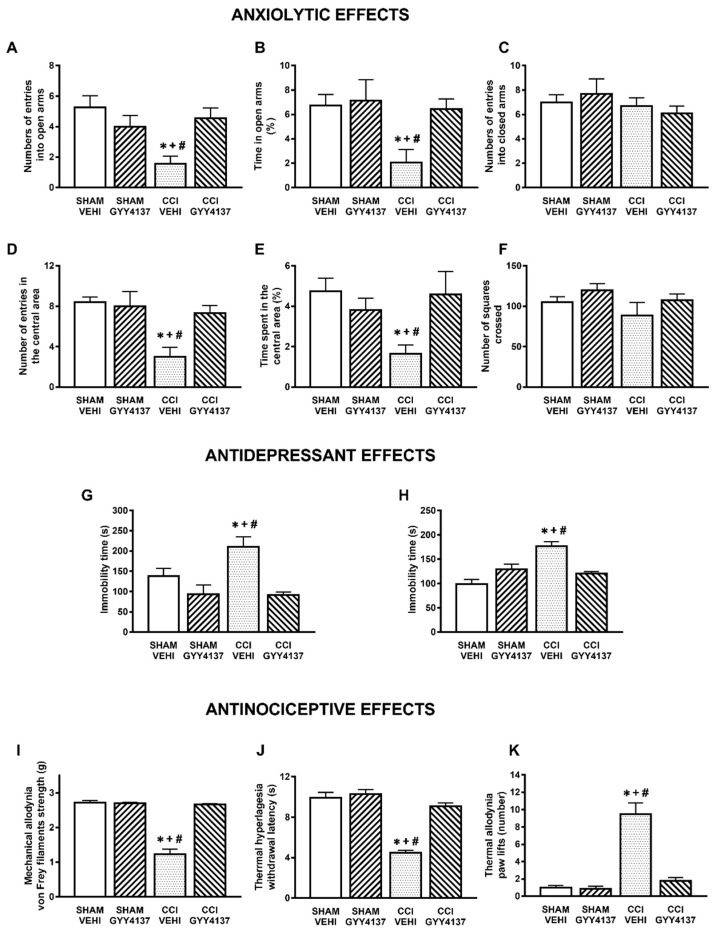
Treatment with GYY4137 inhibited the anxiety- and depressive-like behaviors and the nociceptive responses induced by nerve injury. Effects of the repeated administration with GYY4137 or vehicle at 32 µmol/kg for 4 days, at 2 times for each day, on the anxiety-, depressive-, and nociceptive-like behaviors induced by nerve injury at 30 days after surgery. The effects of GYY4137 or vehicle in sham-operated mice are also displayed. In the EPM test, the number of entries to the open arms (**A**), percentage of the time spent in the open arms (**B**), and the number of entries into the closed arms (**C**) are shown. In the OF test, the number of entries in the central area (**D**), the percentage of time spent in the central area (**E**), and the number of squares crossed (**F**) are presented. In the TST (**G**) and FST (**H**), the immobility times (s) are shown. The effects of GYY4137 in the mechanical allodynia (**I**), thermal hyperalgesia (**J**), and thermal allodynia (**K**) in the ipsilateral paw of sham-operated or sciatic nerve-injured mice are also indicated. For each test: * denotes significant differences vs. sham-operated mice treated with vehicle, + vs. sham-operated mice treated with GYY4137, and # vs. CCI mice treated with GYY4137 (*p* < 0.05; one-way ANOVA followed by the Student–Newman–Keuls test). Mean values ± SEM; *n* = 6–8 animals.

The effects of the repetitive intraperitoneal administration of DADS at 150 µmols/kg, two times a day, during 3 consecutive days and those of GYY4137 administered at 32 µmols/kg, two times a day, during 4 days in the anxiety- and depressive-liked comportments associated with chronic neuropathic pain were evaluated. The results show that both DADS and GYY4137 normalized the diminished number of entries into the open arms (*p* < 0.013; one-way ANOVA followed by the Student–Newman–Keuls test, as compared with their respective sham-operated mice treated with vehicle; Figure 1A and Figure 2A) and the percentage of time spend in its (*p* < 0.008; one-way ANOVA followed by the Student–Newman–Keuls test, as compared with their respective sham-operated mice treated with vehicle; Figure 1B and Figure 2B). No changes in the number of entries into the closed arms were observed (Figure 1C and Figure 2C). We further assessed the effects of DADS and GYY4137 in the OF test to verify their anxiolytic effects during neuropathic pain. The administration of DADS and GYY4137 both normalized the reduced number of entries into the central area (*p* < 0.004; one-way ANOVA followed by the Student–Newman–Keuls test, as compared with sham-operated mice treated with vehicle; Figure 1D and Figure 2D) and the shorter amount of time spent in the central area (*p* < 0.029; one-way ANOVA followed by the Student–Newman–Keuls test, as compared with sham-operated mice treated with vehicle; Figure 1E and Figure 2E), but did not modify the number of squares crossed (Figure 1F and Figure 2F).

Regarding the antidepressant effects, both treatments reduced the high immobility time observed in sciatic nerve-injured animals treated with vehicle in the TST (*p* < 0.001; one-way ANOVA followed by the Student–Newman–Keuls test, as compared with sham-operated mice treated with vehicle; Figure 1G and Figure 2G) and in the FST (*p* < 0.021; one-way ANOVA followed by the Student–Newman–Keuls test, as compared with sham-operated mice treated with vehicle; Figure 1H and Figure 2H), thus revealing the antidepressant effects of these H_2_S donors under neuropathic pain conditions.

Our data also demonstrated the completed reversion of the mechanical allodynia (*p* < 0.001; one-way ANOVA followed by the Student–Newman–Keuls test, as compared with sham-operated mice treated with vehicle) (Figure 1I and Figure 2I), thermal hyperalgesia (*p* < 0.001; one-way ANOVA followed by the Student–Newman–Keuls test, as compared with sham-operated mice treated with vehicle) (Figure 1J and Figure 2J) and cold allodynia (*p* < 0.001; one-way ANOVA followed by the Student–Newman–Keuls test, as compared with sham-operated mice treated with vehicle) (Figure 1K and Figure 2K) provoked by nerve injury in animals treated with DADS or GYY4137 during 3 and 4 days, respectively. The repetitive administration of DADS or GYY4137 did not have any significant effect either in the contralateral paw of sciatic nerve-injured or sham-operated animals (data not shown).

### 3.2. The Acute Administration of DADS and GYY4137 Inhibited Mechanical Allodynia, Thermal Hyperalgesia, and Cold Allodynia Induced by Sciatic Nerve Injury in a Dose-Dependent Manner

**Figure 3:**
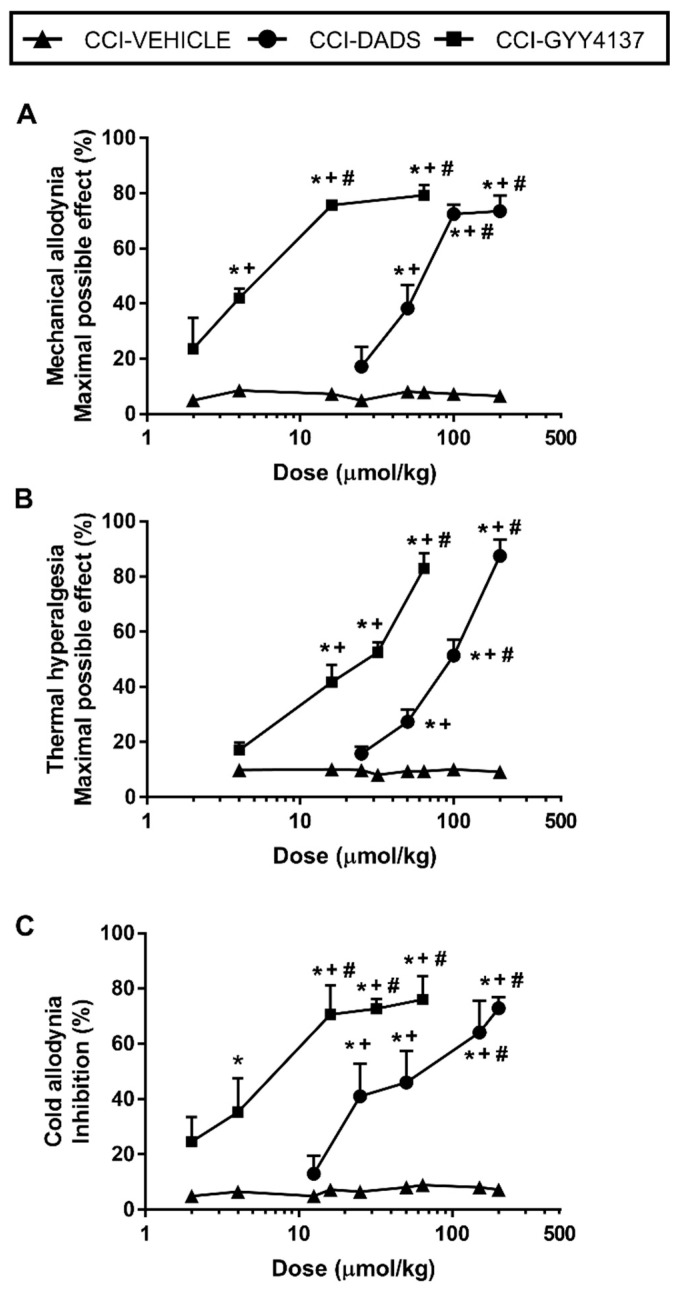
Effects of the acute administration of DADS and GYY4137 in the allodynia and hyperalgesia induced by sciatic nerve injury. Mechanical antiallodynic (**A**), thermal antihyperalgesic (**B**), and thermal antiallodynic effects (**C**) of different doses (logarithmic axis) of DADS and GYY4137 (µmols/kg) are shown. For each dose evaluated, * indicates significant differences vs. animals treated with vehicle, + indicates significant differences vs. the effect produced by the lowest dose of DADS or GYY4137, # vs. the effect produced by other doses of DADS or GYY4137 (*p* < 0.05; one-way ANOVA, followed by Student–Newman–Keuls test). Data are expressed as mean values of maximal possible effect (%) for mechanical allodynia and thermal hyperalgesia and as % inhibition for cold allodynia. Mean values ± SEM (*n* = 6 animals per dose).

The administration of DADS (12.5–200 µmol/kg) or GYY4137 (2–64 µmols/kg) inhibited mechanical allodynia (Figure 3A), thermal hyperalgesia (Figure 3B), and cold allodynia (Figure 3C) incited by sciatic nerve injury in a dose-dependent manner. As a consequence, the mechanical antiallodynic and thermal antihyperalgesic effects produced by high doses of DADS (100, 150 or 200 µmols/kg) were significantly higher than those produced by lower doses (*p* < 0.001; one-way ANOVA). Similarly, the inhibitory effects produced by 16, 32, or 64 µmols/kg of GYY4137 were significantly higher than those produced by lower doses of this drug (*p* < 0.001; one-way ANOVA). In all tests, the effects of DADS at 50, 100, or 200 µmols/kg and GYY4137 at 16, 32, or 64 µmols/kg were greater than those produced by vehicle (*p* < 0.001; one-way ANOVA). Our findings also revealed that DADS or GYY4137 intraperitoneally administered did not have any significant effect either in the ipsilateral paw of sham-operated mice or in the contralateral paw of sciatic nerve-injured or sham-operated animals (data not shown). Regarding the ED_50_, GYY4137 was about 11.8, 7.2 and 9.9 times more effective than DADS in inhibiting mechanical allodynia, thermal hyperalgesia, and cold allodynia, respectively (Table 1).

### 3.3. Reversion of the Antinociceptive Effects of DADS and GYY4137 with the Administration of XE-991 or SnPP

The involvement of H_2_S and HO-1 in the antinociceptive actions of DADS and GYY4137 during neuropathic pain was demonstrated by the reversion of their effects with the selective Kv7 potassium channel blocker, XE-991 (8.0 µmol/kg), and the HO-1 inhibitor, SnPP (14.5 µmol/kg). Our results showed that both XE-991 and SnPP reversed the mechanical anti-allodynic effects produced by 200 µmol/kg of DADS (*p* < 0.001, one-way ANOVA vs. saline + vehicle treated mice) or 64 µmol/kg of GYY4137 (*p* < 0.001, one-way ANOVA vs. saline + vehicle treated mice) (Table 2), in addition to the thermal antihyperalgesic and anti-allodynic effects produced by DADS (*p* < 0.001, one-way ANOVA vs. saline + vehicle treated mice) and GYY4137 (*p* < 0.001, one-way ANOVA vs. saline + vehicle treated mice) in animals with neuropathic pain. The administration of XE-991 and SnPP alone did not produce any significant effect in the ipsilateral and contralateral paws of CCI animals (data not shown).

### 3.4. Effects of Treatment with DADS and GYY4137 in the Protein Levels of PI3K, p-Akt, p-ERK 1/2, CD11b/c, and BAX in the AMG and PAG of Mice with Neuropathic Pain

**Figure 4:**
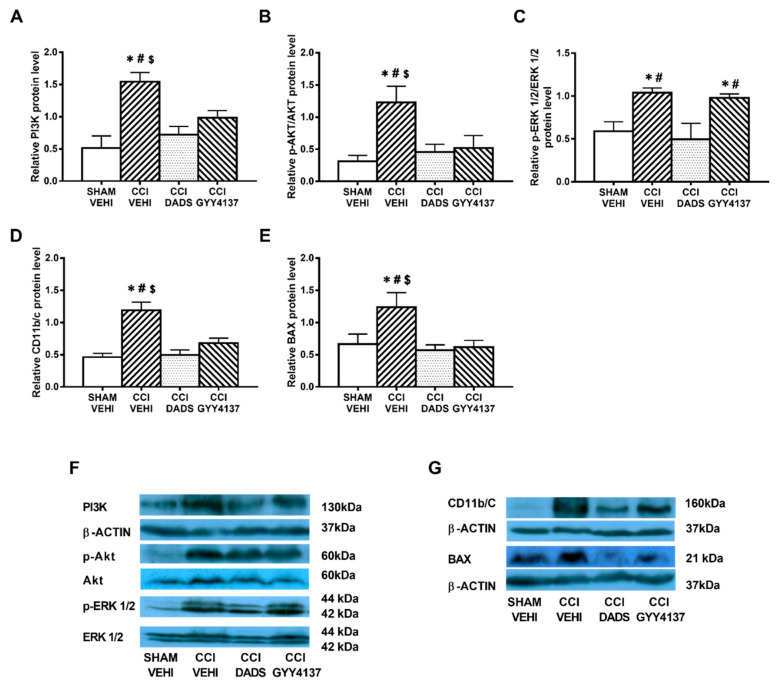
The effects of DADS and GYY4137 on the expression of PI3K, p-AKT, p-ERK 1/2, CD11b/c, and BAX in the AMG of animals with neuropathic pain. Treatment with DADS and GYY4137 normalized the up-regulation of PI3K (**A**), p-AKT (**B**), p-ERK 1/2 (**C**), CD11b/c (**D**), and BAX (**E**) in the AMG of animals with CCI-induced neuropathic pain. We used sham-operated mice treated with vehicle as controls. Non-phosphorylated proteins are expressed relative to β-actin protein levels while phosphorylated proteins are expressed relative to their corresponding total protein levels. Representative blots for PI3K, β-actin, p-Akt/Akt, and p-ERK 1/2/total ERK 1/2 (**F**) and for CD11b/c, BAX, and β-actin (**G**) are shown. In all panels, * represents significant differences vs. sham-operated mice treated with vehicle; # vs. sciatic nerve-injured mice treated with DADS; and $ vs. sciatic nerve-injured mice treated with GYY4137 (*p* < 0.05; one-way ANOVA followed by the Student–Newman–Keuls test). Mean values ± SEM; *n* = 3–4 samples.

**Figure 5:**
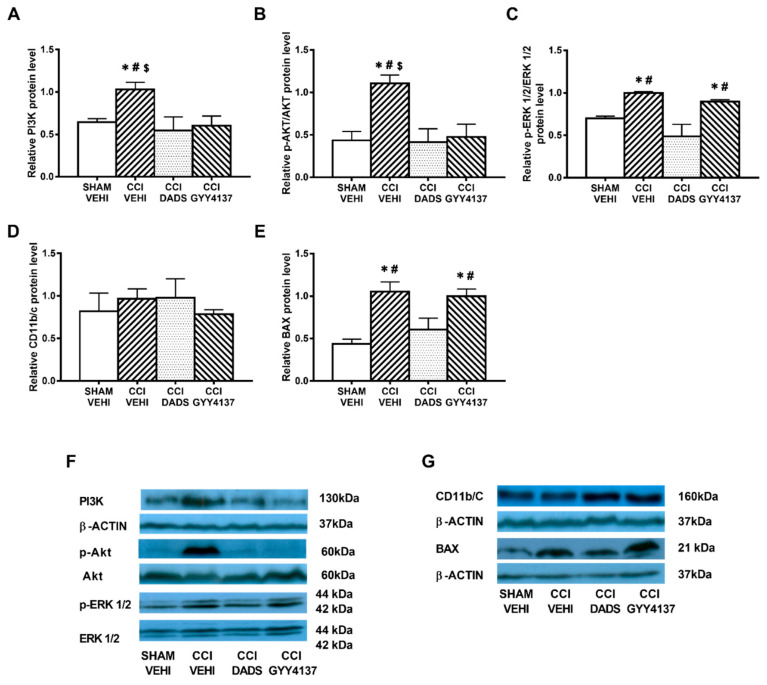
The effects of DADS and GYY4137 on the expression of PI3K, p-AKT, p-ERK 1/2, CD11b/c, and BAX in the PAG of animals with neuropathic pain. Treatment with DADS and/or GYY4137 normalized the up-regulation of PI3K (**A**), p-AKT (**B**), p-ERK 1/2 (**C**), and BAX (**E**) in the PAG of animals with CCI-induced neuropathic pain. No changes in the expression of CD11b/c were observed (**D**). Sham-operated mice treated with vehicle were used as controls. Non-phosphorylated proteins are expressed relative to β-actin protein levels while phosphorylated proteins are expressed relative to their corresponding total protein levels. Representative blots for PI3K, β-actin, p-Akt/Akt, and p-ERK 1/2/total ERK 1/2 (**F**) and for CD11b/c, BAX and β-actin (**G**) are displayed. In all panels, * represents significant differences vs. sham-operated mice treated with vehicle; # vs. sciatic nerve-injured mice treated with DADS; and $ vs. sciatic nerve-injured mice treated with GYY4137 (*p* < 0.05; one-way ANOVA followed by the Student–Newman–Keuls test). Mean values ± SEM; *n* = 3–4 samples.

Sciatic nerve injury caused an up-regulation of PI3K (*p* < 0.032; one-way ANOVA vs. sham-operated vehicle treated mice) (Figure 4A and Figure 5A); p-Akt (*p* < 0.018; one-way ANOVA vs. sham-operated vehicle treated mice) (Figure 4B and Figure 5B), p-ERK 1/2 (*p* < 0.006; one-way ANOVA vs. sham-operated vehicle treated mice) (Figure 4C and Figure 5C) and BAX (*p* < 0.031; one-way ANOVA vs. sham-operated vehicle treated mice) (Figure 4E and Figure 5E) in the AMG and PAG. In both areas, the up regulation of PI3K and p-Akt were reversed with DADS and GYY4137 treatments, whereas p-ERK 1/2 activation was only inhibited with DADS. Moreover, while the up regulation of BAX in the AMG was normalized with DADS and GYY4137, only DADS reversed its up regulation in the PAG. Microglial activation induced by nerve injury in the AMG (*p* < 0.003; one-way ANOVA vs. sham-operated vehicle treated mice) (Figure 4D), but not in the PAG (Figure 5D), was inhibited with both DADS and GYY4137.

### 3.5. Effects of Treatment with DADS and GYY4137 on the Protein Levels of HO-1, NQO1, SOD-1, and GSTM1 in the AMG and PAG of Mice with Neuropathic Pain

**Figure 6:**
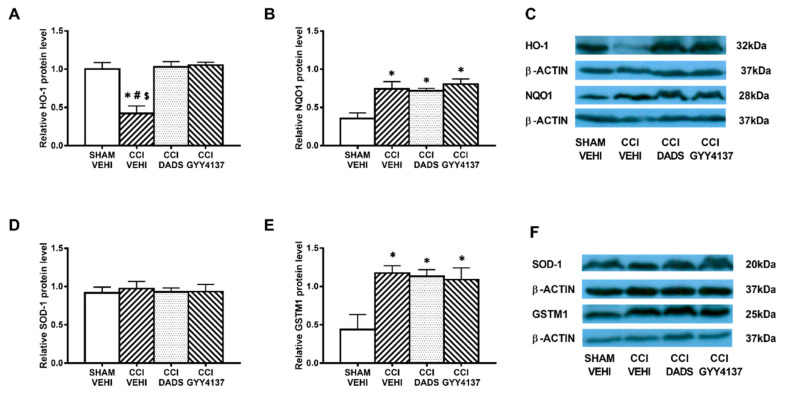
The effects of DADS and GYY4137 on the expression of HO-1, NQO1, SOD-1, and GSTM1 in the AMG of animals with neuropathic pain. Treatment with DADS and GYY4137 normalized and/or increased the protein levels of HO-1 (**A**), NQO1 (**B**), and GSTM1 (**E**) in the AMG of animals with CCI-induced neuropathic pain. No changes in the expression of SOD-1 (**D**) were detected. We used sham-operated mice treated with vehicle as controls. Proteins are expressed relative to β-actin protein levels. Representative blots for HO-1, NQO1, and β-actin (**C**) and for SOD-1, GSTM1, and β-actin (**F**) are shown. In all panels, * represents significant differences vs. sham-operated mice treated with vehicle; # vs. sciatic nerve-injured mice treated with DADS; and $ vs. sciatic nerve-injured mice treated with GYY4137 (*p* < 0.05; one-way ANOVA followed by the Student–Newman–Keuls test). Mean values ± SEM; *n* = 3–4 samples.

**Figure 7:**
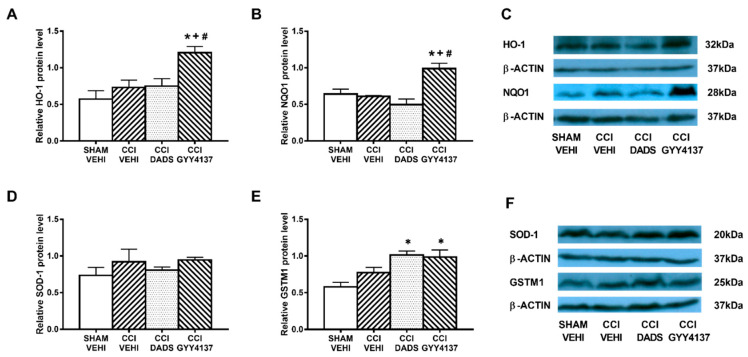
The effects of DADS and GYY4137 on the expression of HO-1, NQO1, SOD-1, and GSTM1 in the PAG of animals with neuropathic pain. Treatment with GYY4137 and/or DADS increased the protein levels of HO-1 (**A**), NQO1 (**B**), and GSTM1 (**E**) in the PAG of animals with CCI-induced neuropathic pain. No changes in the expression of SOD-1 (**D**) were observed. We used sham-operated mice treated with vehicle as controls. Proteins are expressed relative to β-actin protein levels. Representative blots for HO-1, NQO1, and β-actin (**C**) and for SOD-1, GSTM1, and β-actin (**F**) are displayed. In all panels, * represents significant differences vs. sham-operated mice treated with vehicle; + vs. sciatic nerve-injured mice treated with vehicle; and # vs. sciatic nerve-injured mice treated with DADS (*p* < 0.05; one-way ANOVA followed by the Student–Newman–Keuls test). Mean values ± SEM; *n* = 3–4 samples.

In both brain areas, we further evaluated the effects of DADS and GYY4137 in expression of the antioxidant proteins HO-1, NQO1, SOD-1, and GSTM1. Decreased expression of HO-1 (*p* < 0.003; one-way ANOVA vs. sham-operated vehicle treated mice) (Figure 6A) and increased levels of NQO1 (*p* < 0.003; one-way ANOVA vs. sham-operated vehicle treated mice) (Figure 6B) and GSTM1 (*p* < 0.018; one-way ANOVA vs. sham-operated vehicle treated mice) (Figure 6E) were demonstrated in the AMG of sciatic nerve-injured mice. Moreover, both DADS and GYY4137 treatments normalized the decreased expression of HO-1 (Figure 6A) and maintained the elevated levels of NQO1 (Figure 6B) and GSTM1 (Figure 6E) in the AMG. In the PAG, GYY4137 improved the protein levels of HO-1 (*p* < 0.004; one-way ANOVA vs. sham-operated and CCI vehicle treated mice) (Figure 7A) and NQO1 (*p* < 0.003; one-way ANOVA vs. sham-operated and CCI vehicle treated mice) (Figure 7B). Regarding GSTM1, both treatments improved its expression in the PAG (*p* < 0.009; one-way ANOVA vs. sham-operated vehicle treated mice) (Figure 7E). No changes in the expression of SOD-1 were manifested either in the AMG (Figure 6D) or in the PAG (Figure 7D) of animals with sciatic nerve injury.

## 4. Discussion

This study demonstrated the anxiolytic and antidepressant effects of DADS and GYY4137 in mice with chronic neuropathic pain and that GYY4137 is more potent than DADS in inhibiting the allodynia and hyperalgesia provoked by sciatic nerve injury. These activities are mainly produced via inhibiting the PI3K/p-AKT and p-ERK 1/2 up-regulation, microglial activation, and apoptotic responses provoked by nerve injury as well as by modulating the endogenous antioxidant system in the AMG and/or PAG. Both treatments mediated their antinociceptive effects via activating the Kv7 potassium channels and the HO-1 signaling pathway.

It is well known that chronic pain concurs with several emotional disorders, which treatment has not completely resolved. This study demonstrates, for the first time, the inhibition of the anxiolytic and depressive-like behaviors induced by DADS and GYY4137 during neuropathic pain. These results are consistent with the anxiolytic and antidepressant actions of other H_2_S donors, Na_2_S, NaHS, and garlic, in different animal models of anxiety and depression as well as in diabetic rats with depressive-like behaviors [7,10,11]. Our results further confirmed the antidepressant properties of other slow H_2_S releasers such as several isothiocyanates in animals with depressive-like behaviors but were in contrast to the lack of anxiolytic effects of these compounds during chronic osteoarthritic or neuropathic pain [25,43]. These dissimilar effects might be probably related with the different chemical structure of isothiocyanates vs. natural garlic derivates (DADS) or the synthetic H_2_S donor (GYY4137). In relation to other known compounds that also have an impact on the emotional component such as gabapentinoids and several antidepressants [44,45,46], it is important to emphasize that although some of them can inhibit neuropathic pain and improve the associated anxiety-like behaviors, for example, gabapentin and pregabalin, they did not modify the depressive-like behaviors [47,48]. In contrast, different antidepressants, such as duloxetine, decreased pain hypersensitivity and depression-like behaviors in animals with CCI-induced neuropathic pain [49], but did not inhibit the anxiety-like behaviors [47]. In accordance with our results, several antidepressants for, instance, imipramine, milnacipran, and paroxetine, in addition to attenuating the nociceptive responses also have anxiolytic actions during neuropathic pain [50].

In summary, our data revealed, for the first time, the effectivity of DADS and GYY4137 in reducing emotional disorders (anxiety- and depressive-like behaviors) accompanying chronic neuropathic pain. Moreover, and in accordance with other studies [11,51], our findings reported the lack effects of DADS and GYY4137 in the locomotor activity of CCI-injured mice, thus showing the low side effects induced by both treatments during neuropathic pain.

Microglial activation and oxidative stress play an important role in the control of the anxiety and depressive-like behaviors [52]. In accordance, microglial activation has been shown in different brain regions of depressive patients suffering chronic pain [16] and in the hippocampus of animals with anxiety- and depressive-like behaviors associated with neuropathic pain [15,24]. Our data further demonstrate that nerve injury also activates microglia in the AMG, thus reinforcing the key role played by this brain area in the control of the affective components of neuropathic pain [30,53]. The normalization of the overexpression of CD11b/c induced by DADS and GYY4137 suggests the participation of microglia in their anxiolytic and antidepressant effects in CCI-injured mice.

Sciatic nerve ligation also produces important neuroplastic changes in the brain, which further contribute to the development of the anxiety- and depressive-like behaviors present in prolonged pain syndromes [9]. Our results reinforced these findings by showing increased p-ERK 1/2 levels in the AMG, thus supporting the correlation between ERK activation and the depressive-like behaviors observed in sciatic nerve-injured mice [53]. The inhibition of ERK activation induced by DADS might also contribute to its antidepressant and/or anxiolytic actions.

Depression and anxiety disorders are also related to an imbalance between ROS and antioxidant enzyme levels [54,55,56]. Therefore, animals with oxidative stress in the hippocampus and prefrontal cortex [25] or in the peripheral blood granulocytes exhibited signs of anxiety- and depressive-like behaviors [57]. Interestingly, DADS and GYY4137 both avoided the decreased expression of HO-1 and maintained the elevated protein levels of NQO1 and GSTM1 in the AMG, revealing that the potent antioxidant actions of these compounds might also take part in the attenuation of the anxiety- and depressive-like behaviors induced by both compounds during neuropathic pain. Similarly, other authors revealed that garlic and different organosulfur compounds of garlic also potentiated the synthesis of antioxidant enzymes such as glutathione peroxidase in diabetic animals [58] and that the effectivity of GYY4137 against several neurological diseases is mainly accomplished via activating HO-1 synthesis [51,59,60].

Our data further revealed that DADS and GYY4137 inhibited the mechanical and cold allodynia, and thermal hyperalgesia induced by CCI in a dose-dependent manner. These results agree with the proven analgesic effects of GYY4137 in the neuropathy- induced by chemotherapeutic agents [39,41] and further reported the potential pain reliever actions of both DADS and GYY4137 treatments under neuropathic pain conditions generated by nerve injury. Regarding their effectiveness, our data showed that GYY4137 is 11.8 times more potent that DADS in inhibiting the mechanical allodynia and between 9.9 and 7.2 times more effective in decreasing cold allodynia and thermal hyperalgesia induced by CCI, respectively. Our findings reinforce the hypothesis that systemic administration of H_2_S slow-release agents is particularly effective in relieving chronic pain [61,62] and reveal the greater efficacy of synthetic (GYY4137) versus natural H_2_S donors (DADS). Moreover, we also demonstrate that the relief of neuropathic pain induced by DADS and GYY4137 was mediated via activation of the voltage gated Kv7 potassium channels as validated with the blockage of their antiallodynic and antihyperalgesic effects with the Kv7 potassium channel blocker, XE-991 [41].

In this study, we also demonstrated the neuroprotective properties of DADS and GYY4137 in animals with neuropathic pain with the reversion of the enhanced BAX levels induced by sciatic nerve-injury in the AMG and PAG. These results agree with the protective effects of DADS in neuronal cells against apoptosis both in vitro [63] and in vivo [64]. The neuroprotective effects of DADS and GYY4137 also participate in the painkiller actions of these compounds.

It is well known that PAG plays an important role in the descending modulation of pain [65] and the PI3K/p-Akt and ERK activation induced by nerve injury in this area supported the significant role played by these proteins during the maintenance of neuropathic pain [26]. The inhibition of these pathways with DADS and/or GYY4137 treatments revealed that their antinociceptive effects are mainly mediated via PAG regulation. In addition, the antioxidant effects of GYY4137 and DADS in this brain section proved by the upregulation of the expression of HO-1, NQO1, and GSTM1 might also contribute to the relief of chronic pain. Moreover, the reversal of the analgesic effects of DADS and GYY4137 with SnPP (an HO-1 inhibitor) suggest that the slow releasing H_2_S donors alleviate neuropathic pain by activating the HO-1 signaling pathway. For the first time, we have demonstrated the participation of HO-1 in the antinociceptive effects of these treatments, and we postulate a positive interaction between H_2_S and carbon monoxide systems under neuropathic pain conditions.

## 5. Conclusions

In summary, our results reveal the anxiolytic and antidepressant effects of GYY4137 and DADS during neuropathic pain as well as their analgesic properties by blocking the nociceptive, microglial, and apoptotic responses and activating the antioxidant system in the AMG and PAG. Thus, we suggest the potential use of these treatments, especially GYY4137, as desirable candidates for the management of affective disorders accompanying chronic neuropathic pain.

## Figures and Tables

**Table 1:** Comparison of the potencies (ED_50_) of DADS and GYY4137 in the inhibition of the mechanical allodynia, thermal hyperalgesia, and thermal allodynia induced by sciatic nerve injury. Data are expressed as ED_50_ values (µmol/Kg) ± SEM (*n* = 6 animals per dose). For each test, the ratio of the ED_50_ values between drugs is also indicated.

Treatments	Mechanical Allodynia	Thermal Hyperalgesia	Thermal Allodynia
DADS	54.1 ± 8.1	186.5 ± 22.5	60.3 ± 6.4
GYY4137	4.6 ± 1.7	25.9 ± 6.0	6.1 ± 3.4
Ratio (DADS/GYY4137)	11.8	7.2	9.9

**Table 2:** Reversal of the effects produced by the acute intraperitoneal administration of 200 µmol/kg of DADS or 64 µmols/kg of GYY4137 with 8 µmols/kg of XE-911 or 14.5 µmol/kg of SnPP in the inhibition of the mechanical allodynia, thermal hyperalgesia and thermal allodynia induced by sciatic nerve injury in the ipsilateral paw. Data are expressed as mean ± SEM (*n* = 6 animals per treatment). In each test, * denotes significant differences vs. saline + vehicle treated animals (*p* < 0.05, one-way ANOVA, followed by the Student–Newman–Keuls test).

Treatments	Mechanical Allodynia	Thermal Hyperalgesia	Thermal Allodynia
Saline + vehicle	7.8 ± 1.1	9.4 ± 1.5	5.8 ± 1.3
DADS + vehicle	73.6 ± 5.7 *	81.1 ± 4.4 *	74.8 ± 5.0 *
DADS + XE-911	7.3 ± 4.9	5.5 ± 2.1	9.4 ± 5.6
DADS + SnPP	6.5 ± 4.3	4.5 ± 0.7	14.3 ± 6.4
GYY4137 + vehicle	79.3 ± 3.1 *	83.0 ± 5.6 *	76.0 ± 5.0 *
GYY4137 + XE-911	13.8 ± 7.0	7.2 ± 4.2	24.7 ± 10.1
GYY4137 + SnPP	6.9 ± 4.1	5.0 ± 3.2	12.0 ± 8.2
Saline + XE-911	10.2 ± 6.6	4.4 ± 2.8	15.2 ± 9.7
Saline + SnPP	10.0 ± 4.3	3.6 ± 1.9	12.1 ± 7.6

## Data Availability

Data is contained within the article.

## References

[1] Murphy D., Lester D., Smither F.C., Balakhanlou E. (2020). Peripheral neuropathic pain. NeuroRehabilitation.

[2] Fonseca-Rodrigues D., Amorim D., Almeida A., Pinto-Ribeiro F. (2021). Emotional and cognitive impairments in the peripheral nerve chronic constriction injury model (CCI) of neuropathic pain: A systematic review. Behav. Brain Res..

[3] Predmore B.L., Lefer D.J., Gojon G. (2012). Hydrogen sulfide in biochemistry and medicine. Antioxid. Redox Signal..

[4] Kimura H., Shibuya N., Kimura Y. (2012). Hydrogen sulfide is a signaling molecule and a cytoprotectant. Antioxid. Redox Signal..

[5] Szabo C., Papapetropoulos A. (2017). International Union of Basic and Clinical Pharmacology. CII: Pharmacological Modulation of H2S Levels: H2S Donors and H2S Biosynthesis Inhibitors. Pharmacol. Rev..

[6] Chen W.L., Xie B., Zhang C., Xu K.L., Niu Y.Y., Tang X.Q., Zhang P., Zou W., Hu B., Tian Y. (2013). Antidepressant-like and anxiolytic-like effects of hydrogen sulfide in behavioral models of depression and anxiety. Behav. Pharmacol..

[7] Donatti A.F., Soriano R.N., Leite-Panissi C.R., Branco L.G., de Souza A.S. (2017). Anxiolytic-like effect of hydrogen sulfide (H2S) in rats exposed and re-exposed to the elevated plus-maze and open field tests. Neurosci. Lett..

[8] Tang Z.J., Zou W., Yuan J., Zhang P., Tian Y., Xiao Z.F., Li M.H., Wei H.J., Tang X.Q. (2015). Antidepressant-like and anxiolytic-like effects of hydrogen sulfide in streptozotocin-induced diabetic rats through inhibition of hippocampal oxidative stress. Behav. Pharmacol..

[9] Liu S.Y., Li D., Zeng H.Y., Kan L.Y., Zou W., Zhang P., Gu H.F., Tang X.Q. (2017). Hydrogen Sulfide Inhibits Chronic Unpredictable Mild Stress-Induced Depressive-Like Behavior by Upregulation of Sirt-1: Involvement in Suppression of Hippocampal Endoplasmic Reticulum Stress. Int. J. Neuropsychopharmacol..

[10] Rahmani G., Farajdokht F., Mohaddes G., Babri S., Ebrahimi V., Ebrahimi H. (2020). Garlic (Allium sativum) improves anxiety- and depressive-related behaviors and brain oxidative stress in diabetic rats. Arch. Physiol. Biochem..

[11] Huang Y.J., Lu K.H., Lin Y.E., Panyod S., Wu H.Y., Chang W.T., Sheen L.Y. (2019). Garlic essential oil mediates acute and chronic mild stress-induced depression in rats via modulation of monoaminergic neurotransmission and brain-derived neurotrophic factor levels. Food Funct..

[12] Severino B., Corvino A., Fiorino F., Luciano P., Frecentese F., Magli E., Saccone I., Di Vaio P., Citi V., Calderone V. (2018). 1,2,4-Thiadiazolidin-3,5-diones as novel hydrogen sulfide donors. Eur. J. Med. Chem..

[13] Nin D.S., Idres S.B., Song Z.J., Moore P.K., Deng L.W. (2020). Biological Effects of Morpholin-4-Ium 4 Methoxyphenyl (Morpholino) Phosphinodithioate and Other Phosphorothioate-Based Hydrogen Sulfide Donors. Antioxid. Redox Signal..

[14] Liu W., Lv Y., Ren F. (2018). PI3K/Akt Pathway is Required for Spinal Central Sensitization in Neuropathic Pain. Cell Mol. Neurobiol..

[15] Díaz A.F., Polo S., Gallardo N., Leánez S., Pol O. (2019). Analgesic and Antidepressant Effects of Oltipraz on Neuropathic Pain in Mice by Modulating Microglial Activation. J. Clin. Med..

[16] Loggia M.L., Chonde D.B., Akeju O., Arabasz G., Catana C., Edwards R.R., Hill E., Hsu S., Izquierdo-Garcia D., Ji R.R. (2015). Evidence for brain glial activation in chronic pain patients. Brain.

[17] Sawada A., Niiyama Y., Ataka K., Nagaishi K., Yamakage M., Fujimiya M. (2014). Suppression of bone marrow-derived microglia in the amygdala improves anxiety-like behavior induced by chronic partial sciatic nerve ligation in mice. Pain.

[18] Salim S. (2017). Oxidative Stress and the Central Nervous System. J. Pharmacol. Exp. Ther..

[19] Chen M., Pritchard C., Fortune D., Kodi P., Grados M. (2020). Hydrogen sulfide: A target to modulate oxidative stress and neuroplasticity for the treatment of pathological anxiety. Expert Rev. Neurother..

[20] Ferreira-Chamorro P., Redondo A., Riego G., Pol O. (2021). Treatment with 5-fluoro-2-oxindole Increases the Antinociceptive Effects of Morphine and Inhibits Neuropathic Pain. Cell. Mol. Neurobiol..

[21] Fu E.S., Zhang Y.P., Sagen J., Candiotti K.A., Morton P.D., Liebl D.J., Bethea J.R., Brambilla R. (2010). Transgenic inhibition of glial NF-kappa B reduces pain behavior and inflammation after peripheral nerve injury. Pain.

[22] Guo J.R., Wang H., Jin X.J., Jia D.L., Zhou X., Tao Q. (2017). Effect and mechanism of inhibition of PI3K/Akt/mTOR signal pathway on chronic neuropathic pain and spinal microglia in a rat model of chronic constriction injury. Oncotarget.

[23] Redondo A., Riego G., Pol O. (2020). The Antinociceptive, Antioxidant and Anti-Inflammatory Effects of 5-Fluoro-2-Oxindole during Inflammatory Pain. Antioxidant.

[24] Ferreira-Chamorro P., Redondo A., Riego G., Leánez S., Pol O. (2018). Sulforaphane Inhibited the Nociceptive Responses, Anxiety- and Depressive-Like Behaviors Associated with Neuropathic Pain and Improved the Anti-allodynic Effects of Morphine in Mice. Front. Pharmacol..

[25] Cabarga L., Batallé G., Pol O. (2020). Treatment with slow-releasing hydrogen sulfide donors inhibits the nociceptive and depressive-like behaviours accompanying chronic neuropathic pain: Endogenous antioxidant system activation. J. Psychopharmacol..

[26] Mor D., Bembrick A.L., Austin P.J., Keay K.A. (2011). Evidence for cellular injury in the midbrain of rats following chronic constriction injury of the sciatic nerve. J. Chem. Neuroanat..

[27] Afrazi S., Esmaeili-Mahani S., Sheibani V., Abbasnejad M. (2014). Neurosteroid allopregnanolone attenuates high glucose-induced apoptosis and prevents experimental diabetic neuropathic pain: In vitro and in vivo studies. J. Steroid. Biochem. Mol. Biol..

[28] LeDoux J. (2003). The emotional brain, fear, and the amygdala. Cell Mol. Neurobiol..

[29] Veinante P., Yalcin I., Barrot M. (2013). The amygdala between sensation and affect: A role in pain. J. Mol. Psychiatry..

[30] Ikeda R., Takahashi Y., Inoue K., Kato F. (2007). NMDA receptor-independent synaptic plasticity in the central amygdala in the rat model of neuropathic pain. Pain.

[31] Sun Y., Wang J., Liang S.H., Ge J., Lu Y.C., Li J.N., Chen Y.B., Luo D.S., Li H., Li Y.Q. (2020). Involvement of the Ventrolateral Periaqueductal Gray Matter-Central Medial Thalamic Nucleus-Basolateral Amygdala Pathway in Neuropathic Pain Regulation of Rats. Front. Neuroanat..

[32] Hervera A., Leánez S., Motterlini R., Pol O. (2013). Treatment with carbon monoxide-releasing molecules and an HO-1 inducer enhances the effects and expression of µ-opioid receptors during neuropathic pain. Anesthesiology.

[33] Chaplan S.R., Bach F.W., Pogrel J.W., Chung J.M., Yaksh T.L. (1994). Quantitative assessment of tactile allodynia in the rat paw. J. Neurosci. Methods.

[34] Hargreaves K., Dubner R., Brown F., Flores C., Joris J. (1988). A new and sensitive method for measuring thermal nociception in cutaneous hyperalgesia. Pain.

[35] Walf A.A., Frye C.A. (2007). The use of the elevated plus maze as an assay of anxiety-related behavior in rodents. Nat. Protoc..

[36] Sturman O., Germain P.L., Bohacek J. (2018). Exploratory rearing: A context- and stress-sensitive behavior recorded in the open-field test. Stress.

[37] Steru L., Chermat R., Thierry B., Simon P. (1985). The tail suspension test: A new method for screening antidepressants in mice. Psychopharmacology.

[38] Porsolt R.D., Le Pichon M., Jalfre M. (1977). Depression: A new animal model sensitive to antidepressant treatments. Nature.

[39] Qabazard B., Masocha W., Khajah M., Phillips O.A. (2020). H2S donor GYY4137 ameliorates paclitaxel-induced neuropathic pain in mice. Biomed. Pharmacother..

[40] Blackburn-Munro G., Jensen B.S. (2003). The anticonvulsant retigabine attenuates nociceptive behaviours in rat models of persistent and neuropathic pain. Eur. J. Pharmacol..

[41] Mannelli L.D.C., Lucarini E., Micheli L., Mosca I., Ambrosino P., Soldovieri M.V., Martelli A., Testai L., Taglialatela M., Calderone V. (2017). Effects of natural and synthetic isothiocyanate-based H2S-releasers against chemotherapy-induced neuropathic pain: Role of Kv7 potassium channels. Neuropharmacology.

[42] Castany S., Carcolé M., Leánez S., Pol O. (2016). The role of carbon monoxide on the anti-nociceptive effects and expression of cannabinoid 2 receptors during painful diabetic neuropathy in mice. Psychopharmacology.

[43] Batallé G., Cabarga L., Pol O. (2020). The Inhibitory Effects of Slow-Releasing Hydrogen Sulfide Donors in the Mechanical Allodynia, Grip Strength Deficits, and Depressive-Like Behaviors Associated with Chronic Osteoarthritis Pain. Antioxidants.

[44] Bymaster F.P., Lee T.C., Knadler M.P., Detke M.J., Iyengar S. (2005). The dual transporter inhibitor duloxetine: A review of its preclinical pharmacology, pharmacokinetic profile, and clinical results in depression. Curr. Pharm. Des..

[45] Micó J.A., Prieto R. (2012). Elucidating the mechanism of action of pregabalin: α(2)δ as a therapeutic target in anxiety. CNS Drugs..

[46] Navarrete F., Pérez-Ortiz J.M., Manzanares J. (2012). Pregabalin- and topiramate-mediated regulation of cognitive and motor impulsivity in DBA/2 mice. Br. J. Pharmacol..

[47] Grégoire S., Michaud V., Chapuy E., Eschalier A., Ardid D. (2012). Study of emotional and cognitive impairments in mononeuropathic rats: Effect of duloxetine and gabapentin. Pain.

[48] La Porta C., Lara-Mayorga I.M., Negrete R., Maldonado R. (2016). Effects of pregabalin on the nociceptive, emotional and cognitive manifestations of neuropathic pain in mice. Eur. J. Pain.

[49] Hu B., Doods H., Treede R.D., Ceci A. (2016). Duloxetine and 8-OH-DPAT, but not fluoxetine, reduce depression-like behaviour in an animal model of chronic neuropathic pain. Neurosci. Lett..

[50] Matsuzawa-Yanagida K., Narita M., Nakajima M., Kuzumaki N., Niikura K., Nozaki H., Takagi T., Tamai E., Hareyama N., Terada M. (2008). Usefulness of antidepressants for improving the neuropathic pain-like state and pain-induced anxiety through actions at different brain sites. Neuropsychopharmacology.

[51] Yin L., Gao S., Li C. (2020). Exogenous hydrogen sulfide alleviates surgery-induced neuroinflammatory cognitive impairment in adult mice by inhibiting NO signaling. BMC Anesthesiol..

[52] Yirmiya R., Rimmerman N., Reshef R. (2015). Depression as a microglial disease. Trends Neurosci..

[53] Carrasquillo Y., Gereau R.W. (2007). Activation of the extracellular signal-regulated kinase in the amygdala modulates pain perception. J. Neurosci..

[54] Rammal H., Bouayed J., Younos C., Soulimani R. (2008). Evidence that oxidative stress is linked to anxiety-related behaviour in mice. Brain Behav. Immun..

[55] Krolow R., Arcego D.M., Noschang C., Weis S.N., Dalmaz C. (2014). Oxidative imbalance and anxiety disorders. Curr. Neuropharmacol..

[56] Balmus I.M., Ciobica A., Antioch I., Dobrin R., Timofte D. (2016). Oxidative Stress Implications in the Affective Disorders: Main Biomarkers, Animal Models Relevance, Genetic Perspectives, and Antioxidant Approaches. Oxid. Med. Cell. Longev..

[57] Bouayed J., Rammal H., Younos C., Soulimani R. (2007). Positive correlation between peripheral blood granulocyte oxidative status and level of anxiety in mice. Eur. J. Pharmacol..

[58] Drobiova H., Thomson M., Al-Qattan K., Peltonen-Shalaby R., Al-Amin Z., Ali M. (2011). Garlic increases antioxidant levels in diabetic and hypertensive rats determined by a modified peroxidase method. Evid. Based Complement. Alternat. Med..

[59] Zeng T., Zhang C.L., Song F.Y., Zhao X.L., Yu L.H., Zhu Z.P., Xie K.Q. (2013). The activation of HO-1/Nrf-2 contributes to the protective effects of diallyl disulfide (DADS) against ethanol-induced oxidative stress. Biochim. Biophys. Acta.

[60] Xu X., Hu P., Ma Y., Tong L., Wang D., Wu Y., Chen Z., Huang C. (2020). Identification of a pro-elongation effect of diallyl disulfide, a major organosulfur compound in garlic oil, on microglial process. J. Nutr. Biochem..

[61] Kida K., Marutani E., Nguyen R.K., Ichinose F. (2015). Inhaled hydrogen sulfide prevents neuropathic pain after peripheral nerve injury in mice. Nitric Oxide..

[62] Lucarini E., Micheli L., Martelli A., Testai L., Calderone V., Ghelardini C., Di Cesare Mannelli L. (2018). Efficacy of isothiocyanate-based compounds on different forms of persistent pain. J. Pain Res..

[63] García A., Morales P., Arranz N., Delgado M.E., Rafter J., Haza A.I. (2009). Antiapoptotic effects of dietary antioxidants towards N-nitrosopiperidine and N-nitrosodibutylamine-induced apoptosis in HL-60 and HepG2 cells. J. Appl. Toxicol..

[64] He H., Ma Y., Huang H., Huang C., Chen Z., Chen D., Gu Y., Wang X., Chen J. (2021). A comprehensive understanding about the pharmacological effect of diallyl disulfide other than its anti-carcinogenic activities. Eur. J. Pharmacol..

[65] Jaggi A.S., Singh N. (2011). Role of different brain areas in peripheral nerve injury-induced neuropathic pain. Brain Res..

